# Production of Defective Interfering Particles of Influenza A Virus in Parallel Continuous Cultures at Two Residence Times—Insights From qPCR Measurements and Viral Dynamics Modeling

**DOI:** 10.3389/fbioe.2019.00275

**Published:** 2019-10-18

**Authors:** Felipe Tapia, Tanja Laske, Milena A. Wasik, Markus Rammhold, Yvonne Genzel, Udo Reichl

**Affiliations:** ^1^Max Planck Institute for Dynamics of Complex Technical Systems, Magdeburg, Germany; ^2^Chair for Bioprocess Engineering, Otto-von-Guericke-University Magdeburg, Magdeburg, Germany

**Keywords:** influenza A virus, continuous virus production, suspension MDCK cells, defective interfering particles, mathematical model, virus evolution, antiviral

## Abstract

Defective interfering particles (DIPs) are a natural byproduct of influenza A virus (IAV) replication. DIPs interfere with the propagation and spread of infectious standard virus (STV), reduce virus yields by competing for viral and cellular resources, and induce antiviral responses. These properties open exciting possibilities for the development of DIP-based antivirals. Exploring options for cell culture-based DIP production, we have established a fully continuous cultivation process, where one bioreactor is used to grow cells that are fed to two bioreactors operated in parallel for virus production. This system allows head-to-head comparisons of STV and DIP replication dynamics over extended time periods. Cultivations were performed at two residence times (RT, 22 and 36 h) using MDCK suspension cells grown in a fully defined medium. For infection, we used a virus seed generated by reverse genetics containing STVs and a known DIP carrying a deletion in segment 1 (delS1(1)). Four days post infection, DIPs achieved maximum concentrations of 7.0·10^9^ virions/mL and 8.4·10^9^ virions/mL for RTs of 22 and 36 h, respectively. Furthermore, oscillations in virus titers with two to three maxima were found for DIP accumulation at 36 and 22 h RT, respectively. To complement the study, a basic mathematical model using simple kinetics and a reasonable number of parameters to describe DIP-propagation in continuous cultures was established. Upon fitting the model individually to each of the two data sets, oscillations in the viral dynamics and the cell population dynamics were described well. Modeling suggests that both STV inactivation and virus degradation have to be taken into account to achieve good agreement of simulations and experimental data for longer RTs. Together, the high DIP titers obtained, and the successful simulation of the experimental data showed that the combination of continuous bioreactors and mathematical models can enable studies regarding DIP dynamics over extended time periods and allow large scale manufacturing of DIP-based antivirals.

## Introduction

Viruses are a major threat to human health and significant efforts have been made over the last century to prevent and treat viral diseases. A huge success was the development of potent, safe and affordable viral human and veterinary vaccines using egg- and cell-based production systems (Aubrit et al., 2015; Genzel, 2015; Barrett et al., 2017; Volz and Sutter, 2017). In addition, novel approaches toward vaccination are under development, i.e., DNA and RNA vaccines (Kutzler and Weiner, 2008; De Gregorio and Rappuoli, 2014; Ahmed et al., 2017). In addition, for various viruses such as HIV, IAV, or herpes virus, potent antivirals are available for treatment [e.g., for influenza virus (Samson et al., 2013; Simonsen et al., 2018)]. Despite this, significant challenges remain. On the one hand, humans are constantly challenged by new viruses including Zika virus, MERS-coronavirus, or Ebola virus. On the other hand, most viruses change rapidly in the face of selective pressure and emergence of antiviral drug resistance is of major concern [e.g., for influenza virus (Samson et al., 2013; Lackenby et al., 2018; Shin and Seong, 2019)]. These challenges can only partly be addressed by existing technologies, since the development of new vaccines, the adaptation of existing manufacturing processes, and the identification of new antiviral targets are time consuming and costly processes. They involve complex decisions regarding the selection of efficient production systems, potency and safety aspects, and comprehensive clinical trials. Moreover, conventional antiviral measures may be too slow to save lives in case of a rapidly spreading virus.

An unconventional option to prevent virus spreading and cure infectious diseases is the use of viruses themselves. This concerns especially defective interfering particles (DIPs)—defective viruses, which suppress the spread of their intact, replication competent counterparts, reducing infectious virus yields by up to five orders of magnitude (e.g., Akkina et al., 1984; Frensing et al., 2014). DIPs were already identified in the early fifties by studies of von Magnus (von Magnus, 1951), who performed undiluted serial passages of IAV. DIPs are characterized by deletions in the viral genome preventing the synthesis of a protein essential for viral spread (Nayak et al., 1985; Dimmock and Easton, 2015; Frensing, 2015). For replication, DIPs rely on the presence of homologous helper virus, further referred to as standard virus (STV), that supplies the missing viral protein(s) *in trans*. Interestingly, the presence of DIPs has been demonstrated for almost every virus studied (Huang and Baltimore, 1977) making DIPs a viable option to prevent and/or treat a larger number of viral diseases.

While initial studies suggested that DIPs suppress STV replication and may thus have the potential to protect against viral disease, it was not until the late eighties that researchers started to systematically exploit this approach (Dimmock et al., 1986). It was Dimmock and colleagues (Dimmock et al., 2008) who demonstrated that the use of molecular cloning technologies enables the generation of DIPs that have the potential to protect animals from IAV infection. They also showed that the DIP isolate named DI244, from Influenza A/Puerto Rico/8/34 (PR8; H1N1), which contains a single deletion in segment 1 (S1; coding for PB2), is suitable to both therapeutically and prophylactically protect animals from a lethal challenge by the 2009 pandemic IAV and, potentially, other respiratory viruses (Duhaut and Dimmock, 2003; Easton et al., 2011; Scott et al., 2011; Dimmock et al., 2012; Dimmock and Easton, 2014). They showed that production of the DI244 antiviral candidate can be realized in eggs followed by an ultraviolet (UV) irradiation process to destroy STV infectivity (Dimmock et al., 2008). More recently, an *in vitro* and *in vivo* antiviral effect was demonstrated for a combination of three defective interfering genes of IAV for avian and seasonal influenza using a dual-functional peptide vector (Zhao et al., 2018).

Despite the increasing interest in the potential use of DIPs as antiviral agents, relatively little is known regarding their spread and accumulation in cell populations. This also applies to large scale manufacturing of DIPs in biopharmaceutical industry where fertilized chicken eggs or animal cell culture technologies could be considered for efficient large scale DIP production. While eggs have been successfully used for the production of DIPs in relatively small amounts (Dimmock and Marriott, 2006; Dimmock et al., 2008), cell culture-based technologies have been less explored and have several additional advantages for large scale DIP manufacturing. Firstly, animal cells are ideal for in-depth investigation of intracellular DIP replication, their release and cell-to-cell spreading under controlled and well-defined cultivation conditions in bioreactors over an extended time period. Secondly, cells could be specifically designed for DIP generation (Ozawa et al., 2011; Bdeir et al., 2019; Yamagata et al., 2019) using plasmids for reverse genetics (Hoffmann et al., 2000), which would allow to overcome the need of any infectious helper virus for DIP replication. Such DIP preparations, in contrast to egg-based production systems, would not be contaminated with STV and would not need UV inactivation for use as antivirals (Dimmock et al., 2008). Finally, there are various quantitative assays available for detailed characterization of the dynamics of virus titers and DIP copy numbers. Together with the use of specific staining methods and flow cytometry for monitoring the progress of infection in cells (Frensing et al., 2014, 2016; Swick et al., 2014), mathematical models for DIP and STV replication can be established to describe their basic dynamics in cell culture (Frensing et al., 2013; Akpinar et al., 2016a,b; Laske et al., 2016; Liao et al., 2016).

In this study, following the general ideas described by Frensing et al. (2013), we investigated DIP production in a continuous cultivation system. Frensing et al. demonstrated that continuous influenza virus production in a cascade of two stirred tank bioreactors showed oscillations in virus and cell concentrations due to the presence of DIPs. In contrast to their approach using only two vessels, we used one bioreactor for continuous cell production (cell bioreactor or CB) feeding two bioreactors for virus propagation (virus bioreactor 1 or VB1; virus bioreactor 2 or VB2) operated in parallel to allow for head-to-head comparisons of virus seeds, media, cell lines, or changes in cultivation parameters under conditions as close to each other as possible. As a starting point, the impact of residence time (RT, 22 and 36 h) on DIP and STV dynamics was investigated. With regard to the establishment of manufacturing processes for DIP production, a MDCK suspension cell line growing in a fully defined medium was used (Lohr et al., 2010). Virus seeds containing known amounts of DIPs and STV were generated using a reverse genetics approach (Hoffmann et al., 2000). Cultivations were performed over a period of 20 days at two RTs to allow for at least two oscillations in STV and DIP replication dynamics (Frensing et al., 2013). For process monitoring and model development, cell concentrations, infectious and non-infectious virus titers, as well as extracellular copy numbers of S1 for both STV, i.e., full-length (FL) S1, and a known form of DIP, delS1(1), were determined. Based on the newly available quantitative data obtained for DIP and STV concentrations, the mathematical model developed by Frensing et al. (2013) was extended. After fitting, the basic process dynamics for both RTs were described well by the simulations. In particular, these simulations capture the oscillations in process variables, e.g., virus titers. In addition, the model predicts the dynamics of cell subpopulations (uninfected, DIP-only infected, STV-only infected, and co-infected cells).

## Materials and Methods

### Cells and Virus

The canine cell line MDCK.SUS2 (through contact with Prof. Klaus Scharfenberg, University of Applied Sciences Emden-Leer, Germany) was cultivated in chemically defined Smif8 medium (Gibco, Thermo Fischer Scientific, USA), supplemented with glutamine, and pyruvate (both 4 mM final concentration, Sigma, USA). MDCK.SUS2 cells were grown in shake flasks and passaged as described before (Lohr et al., 2010).

Influenza A/Puerto Rico/8/34 virus seed containing DIPs with a deletion in S1, i.e., delS1(1), was generated by reverse genetics using an 8+1 plasmid-system (Hoffmann et al., 2000; Duhaut and Dimmock, 2003). This virus seed will be referred to as A/PR/8/34-delS1(1). Therefore, S1 RNA was isolated from influenza A/Puerto Rico/8/34 (National Institute for Biological Standards and Control (NIBSC), No. 06/114) and the respective cDNA was cloned into the pHW2000 vector provided by Erich Hoffmann and Robert G. Webster from St. Jude Children's Research Hospital (Memphis, TN, USA) (Hoffmann et al., 2000), resulting in the plasmid pHW-S1. The plasmid carrying the defective S1 sequence pHW-A/PR/8/34-delS1(1), previously described by Dimmock et al. (2008), was obtained by cloning the respective sequence into the pHW2000 vector. In addition, pHW plasmids carrying FL segments 2–8 were used (also provided by Erich Hoffmann and Robert G. Webster).

One day before transfection, 5·10^5^ HEK-293T cells were seeded into 35 mm dishes. Prior to infection, cells were washed with PBS and 3 mL Opti-MEM (Gibco) were added. Transfection was performed simultaneously with all nine plasmids, 280 ng each, using Lipofectamine LTX & PLUS Reagent (Life Technologies) according to the manufacturer's instructions (5 μL Lipofectamine LTX per dish). To increase the amount of virus particles, 5·10^5^ adherent MDCK cells were added 7 h post-transfection. After 24 h at 37°C and 5% CO_2_, cells were washed with PBS and medium was replaced with DMEM (Gibco) containing 5 Units/mL trypsin (Gibco, #27250-018, sterile-filtered stock solution prepared in PBS with 500 U/mL and stored at −20°C). Subsequently, the cells were incubated at 37°C for additional 72 h. As a result, a virus seed containing A/PR/8/34-delS1(1) and STV, with a TCID_50_ titer of 5.6·10^5^ virions/mL and an HA titer of 1.91 log_10_(HA Units/100 μL) was obtained. Note, that segment-specific reverse transcription-quantitative PCR (qPCR) to determine A/PR/8/34-delS1(1) copy numbers in virus seeds is biased since it is contaminated with the plasmid carrying the DI S1 sequence, pHW-A/PR/8/34-delS1(1). For low multiplicity of infection (MOI) cultivations in continuous cultures, this is not relevant as plasmids are out-diluted (see below).

In a next step, the virus seed for infection of suspension MDCK cells in bioreactors was generated. Therefore, the A/PR/8/34-delS1(1) virus seed was passaged once in suspension MDCK.SUS2 cells with an MOI of 0.1. The virus was harvested 40 h post infection (p.i.), clarified by centrifugation (1,000 g) and the supernatant was aliquoted. The resulting virus bank had an HA titer of 2.36 log_10_(HA Units/100 μL), a TCID_50_ titer of 6.76·10^7^ virions/mL, a FL S1 content of 9.7·10^9^ copies/mL, and a DI S1 (A/PR/8/34-delS1(1)) content of 2.9·10^8^ copies/mL. Additionally, virus seeds were probed for remaining plasmids after reverse genetic preparation by qPCR, which could bias reverse transcription-qPCR data of the FL and DI S1 measurements. Quantitative PCR data showed, that initial plasmid contamination was 17.6% for A/PR/8/34-delS1(1) and 0.8% for FL S1. However, from zero h p.i. on the plasmid contamination was not detectable any longer, since the virus preparation was diluted due to the low MOI applied for infection (MOI 0.1). All experiments were performed in laboratories with a biological safety level 2 (BSL 2) certification following the respective safety regulations.

### Parallel Bioreactor Setup for Continuous Influenza Virus Propagation

**Figure 1:**
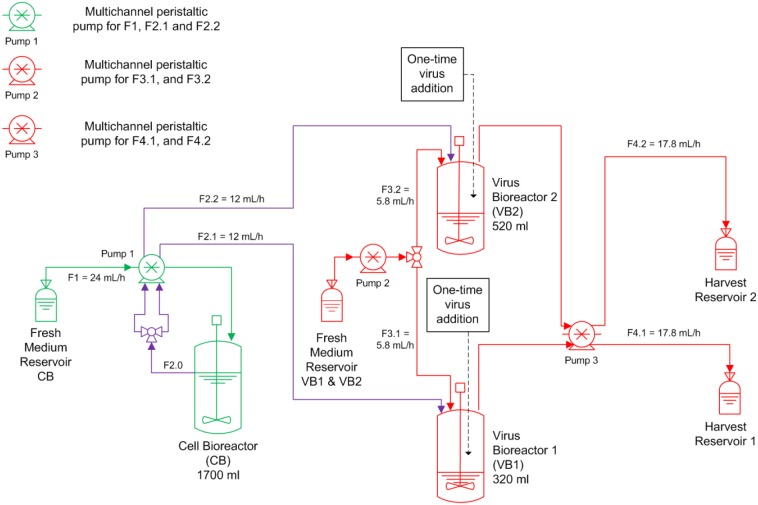
Overview of the bioreactor setup for parallel continuous influenza A virus propagation in two vessels. MDCK suspension cells were grown in a cell bioreactor (CB) feeding two virus production vessels (VB1 and VB2) operated at a residence time of 22 and 36 h, respectively. At time of infection, the suspension MDCK cell-adapted virus seed containing A/PR/8/34-delS1(1) and FL standard virus (STV) was added to VB1 and VB2 at a multiplicity of infection of 0.1. Subsequently, cells were constantly fed into both virus bioreactors (F, feeding rate). Trypsin was added to the fresh medium reservoir of VB1 and VB2. All green components refer to CB, all red components to the VBs; CB and VBs are connected via the purple tubing.

A continuous bioreactor system consisting of three stirred tank bioreactors (STR, Dasgip) was used (Figure 1). The first 1.5 L STR (with head space capacity for 1700 mL working volume) was used for cell propagation (CB), and the other two 0.5 L STR were run in parallel and used for virus propagation (VB1 and VB2). After inoculation with MDCK.SUS2 cells at 1.4·10^6^ viable cells/mL, the CB was operated in batch mode the first 73 h in 1700 mL working volume (wv). Simultaneously, VB1 and VB2 were inoculated with 1.2·10^6^ viable cells/mL and grown in batch mode in 320 mL wv and 520 mL wv, respectively. Working volumes were chosen to achieve RTs of 22 and 36 h in VB1 and VB2, respectively, using the flow rates (F) described in Figure 1 (RT = wv/F). The choice of 22 h RT was motivated by similar experiments performed previously by our group (Frensing et al., 2013). The 36 h RT was selected based on scouting experiments performed in shake flasks and preliminary mathematical simulations (not shown). The CB was maintained at 1700 mL wv. Cultivations parameters were 37 °C, pH 7.1, and a stirring speed of 130 rpm. Aeration was controlled to 30% dissolved oxygen partial pressure by pulsed addition with a mixture of air, oxygen and nitrogen through a dip tube. For infection, the DIP-containing virus seed (A/PR/8/34-delS1(1)) was added to the VB1 and VB2 at an MOI of 0.1 based on the viable cell count and the TCID_50_ titer of the virus seed at 50 h of culture. Additionally, the virus inoculum was supplemented with 1:100 ratio of trypsin (volume to culture volume). To avoid a fast wash-out of the virus seed, continuous mode was initiated at 23.4 h p.i. with the flow rates depicted in Figure 1. During the run, trypsin (0.5 Units/L) was present in the feed medium of the virus bioreactors. Feed media of all three reactors were provided in 5 L bottles chilled at 0-4°C; only the feed medium of the cell bioreactor had to be refilled during the run-time. Samples were taken twice a day from VB1 and VB2 and once a day from CB. About every 12 h, virus harvests reservoirs were stored at−20°C until further analysis. The experiment was terminated 20 days p.i. The average cell concentration input for VB1 and VB2 was 1.13·10^6^ cells/mL as determined from the average viable cell count in CB with respect to STR working volumes and feeding rates. RTs were determined from the average volume and the average flow rate at the outlet of both STR over the infection phase.

### Cell Counts and Virus Titers

Viable cell concentrations were determined using a ViCellXR (Beckman Coulter) and virus production was monitored by a hemagglutination (HA) assay (Kalbfuss et al., 2008) and a TCID_50_ assay (Genzel and Reichl, 2007). The maximum standard deviation of the HA assay was ± 0.15 log_10_(HA Units/100 μL) and the dilution error of the TCID_50_ assay was ± 0.3 log_10_ (Genzel et al., 2014).

Furthermore, we determined the concentration of total virus particles *C*_*V*_ in the supernatant based on the HA values using the following equation,

(1) $$\begin{array}{r} C_{V}=C_{Ery}\cdot 10^{\left({log}_{10}HAU/100\;\mu L\right)}, \end{array}$$

where *C*_*Ery*_ denotes the concentration of chicken erythrocyte solution added to the assay (2 × 10^7^ cells/mL).

### Segment-Specific Reverse Transcription-PCR for Detection of Defective Genomes

To analyze viral genomes, bioreactor samples were centrifuged for 5 min at 300 g and viral RNA was isolated from supernatants using the NucleoSpin RNA Virus kit (Macherey-Nagel) according to the manufacturer's instructions. Reverse transcription of isolated RNA to cDNA and segment-specific amplification of IAV genomes was performed as described previously (Frensing et al., 2014). Reverse transcription-PCR products were directly analyzed on a 1% agarose gel using electrophoresis (see Data Sheet 1; Figure S4).

### Segment-Specific Reverse Transcription-qPCR for A/PR/8/34-delS1(1) and FL S1 Quantification

Absolute viral RNA (vRNA) copy numbers were determined as described previously (Wasik et al., 2018). In brief, 10-fold series dilutions of the corresponding vRNA reference standards and RNA samples were reverse transcribed with the tagged primer Seg-1-tagRT-for (ATTTAGGTGACACTATAGAAGCGAGCGAAAGCAGGTCAATTATATTC). Subsequently, a qPCR was performed using the Rotor-Gene SYBR Green PCR Kit (Qiagen) following the manufacturer's recommendations and the primer pairs realtime-rev (GGAATCCCCTCAGTCTTC) and vRNA-tagRealtime-for (ATTTAGGTGACACTATAGAAGCG) for quantification of A/PR/8/34-delS1(1) and the primer pairs FL1-realtime-rev (CATTTCATCCTAAGTGCTGG) and vRNA-tagRealtime-for for quantification of FL S1. Viral RNA copy numbers were calculated based on the vRNA reference standards with linear regression. The lowest concentrations, which allowed quantification, were 2.2·10^6^ copies/mL for A/PR/8/34-delS1(1) and 3.8·10^6^ copies/mL for FL S1, respectively (Wasik et al., 2018). The relative standard deviation for determining copy numbers by qPCR was 23 and 27% for A/PR/8/34-delS1(1) and FL S1, respectively. Quantification of contaminating plasmids was performed by dilution series of the corresponding plasmid as described above, but without the reverse transcription step. The plasmid pHW-S1 was quantified with the primer pair FL1-realtime-rev and pHW-f (CTCACTATAGGGAGACCC). The plasmid pHW-A/PR/8/34-delS1(1) was quantified with the primer pair realtime-rev and pHW-f.

Since number of genome copies do not directly translate into numbers of virus particles, we adjusted the qPCR measurements of A/PR/8/34-delS1(1) and FL S1. For this, we assumed that the sum of A/PR/8/34-delS1(1)-containing virions and the FL S1-containing virions denotes the maximum number of virions present in a sample, which should equal the concentration of total virus particles *C*_*V*_. Based on *C*_*V*_, we calculated the concentration of virions that contain either A/PR/8/34-delS1(1) or FL S1 using Equations (2) and (3), respectively.

(2) $$\begin{array}{r} V_{ddi}=\frac{delS1\left(1\right)\left(copies/mL\right)}{delS1\left(1\right)+FL\;S1\;\left(copies/mL\right)}\cdot C_{V}\left(virions/mL\right) \end{array}$$

(3) $$\begin{array}{r} V_{S}+V_{d}=\;\frac{FL\;S1\;\left(copies/mL\right)}{delS1\left(1\right)+FL\;S1\;\left(copies/mL\right)}\cdot C_{V}\left(virions/mL\right) \end{array}$$

Furthermore, a differentiation between infectious FL S1-containing virions (*V*_*S*_) and non-infectious FL S1-containing virions (*V*_*d*_) is made. Since we know the number of *V*_*S*_, i.e., the TCID_50_ titer, we can easily determine the number of non-infectious FL S1-containing virions by solving Equation (3) for *V*_*d*_.

### Mathematical Modeling

Based on quantitative data available from reverse transcription-qPCR for S1 encoding PB2 of IAV that was established recently by our group, the segregated mathematical model describing this continuous virus production system established by Frensing et al. (2013) was modified. The extended model version describes explicitly the dynamics of replication-incompetent virions (DIPs) containing a deletion in S1 (*V*_*ddi*_) as described above (A/PR/8/34-delS1(1), see Materials and Methods) in addition to non-infectious virions (*V*_*d*_) and infectious virions (*V*_*s*_) containing the FL S1.

As another modification, the infection of uninfected target cells (*T*) is considered separately for *V*_*s*_ and *V*_*ddi*_ to account for DIP entry with subsequent DIP replication in case of a co-infection.

(4) $$\frac{d\;T}{d\;t}=\mu \;\cdot \;T-kvi\;\cdot \;T\;\cdot \;V_{s}-kvidi\;\cdot \;T\;\cdot \;V_{ddi}+D\;\cdot \;(T_{in}-T)$$

Where μ denotes the specific cell growth rate, and *kvi* and *kvidi* are the specific virus infection rates for infectious STVs and DIPs, respectively. The last term in Equation (4) accounts for the continuous feed of cells with concentration *T*_*in*_ at dilution rate *D*, which was set to adjust the two different RTs for both virus reactors (see Table 1). With respect to the average concentration of cells observed in CB, we choose *T*_*in*_ to be independent of time (data not shown). Ideal mixing is assumed for all vessels.

**Table 1:** Non-zero initial conditions and parameters including coefficients of variation used for simulations.

**Symbol**	**Description**	**Value**	CoV^*^	**Unit**
**Continuous cultivation RT 22 h**				
μ^**^	Maximum spec. cell growth rate	0.0454	*Fixed*	1/*h*
D^**^	Dilution rate of virus reactor	0.0454	*Fixed*	1/*h*
*kvi*	Spec. virus infection rate, infectious STV	1.59·10^−7^	33	*mL*/(*virion*·*h*)
*kvidi*	Spec. virus infection rate, DIP	2.32·10^−10^	59	*mL*/(*virion*·*h*)
*kcdv*	Spec. apoptosis rate	0.008	35	1/*h*
μ*vi*	Spec. *V*_*s*_ production rate	2.51	9	*virions*/(*cell*·*h*)
μ*vddi*_*C*_	Spec. *V*_*ddi*_ production rate of co-infected cells	203	10	*virions*/(*cell*·*h*)
μ*vddi*_*S*_	*De novo* generation of DIPs	1.00·10^−5^	1.40·10^6^	*virions*/(*cell*·*h*)
μ*vd*_*C*_	Spec. *V*_*d*_ production rate of co-infected cells	4.91·10^−16^	4.07·10^15^	*virions*/(*cell*·*h*)
μ*vd*_*S*_	Spec. *V*_*d*_ production rate of STV-infected cells	120	13	*virions*/(*cell*·*h*)
*kdvit*	Spec. *V*_*s*_ inactivation rate	1.58·10^−7^	1.20·10^4^	1/*h*
*kvdt*	Spec. *V*_*ddi*_, *V*_*d*_ lysis rate	3.82·10^−27^	1.15·10^26^	1/*h*
*T*_0_	Initial target cell concentration	1.80·10^6^	−	*cells*/*mL*
*T*_*in*_	Cell concentration in feed	1.13·10^6^	−	*cells*/*mL*
*V*_*s*0_	Initial infectious STV concentration	3.16·10^5^	−	*virions*/*mL*
*V*_*ddi*0_	Initial DIP concentration	6.41·10^5^	−	*virions*/*mL*
*V*_*d*0_	Initial non-infectious STV concentration	2.05·10^7^	−	*virions*/*mL*
**Continuous cultivation RT 36 h**				
μ^**^	Maximum spec. cell growth rate	0.0278	*Fixed*	1/*h*
D^**^	Dilution rate of virus reactor	0.0278	*Fixed*	1/*h*
*kvi*	Spec. virus infection rate, infectious STV	5.38·10^−8^	15	*mL*/(*virion*·*h*)
*kvidi*	Spec. virus infection rate, DIP	7.96·10^−11^	96	*mL*/(*virion*·*h*)
*kcdv*	Spec. apoptosis rate	0.003	80	1/*h*
μ*vi*	Spec. *V*_*s*_ production rate	4.12	25	*virions*/(*cell*·*h*)
μ*vddi*_*C*_	Spec. *V*_*ddi*_ production rate	177	7	*virions*/(*cell*·*h*)
μ*vddi*_*S*_	*De novo* generation of DIPs	1.13·10^−9^	1.12·10^4^	*virions*/(*cell*·*h*)
μ*vd*_*C*_	Spec. *V*_*d*_ production rate of co-infected cells	1.41·10^−8^	3.30·10^10^	*virions*/(*cell*·*h*)
μ*vd*_*S*_	Spec. *V*_*d*_ production rate of STV-infected cells	173	9	*virions*/(*cell*·*h*)
*kdvit*	Spec. *V*_*s*_ inactivation rate	0.070	47	1/*h*
*kvdt*	spec. *V*_*ddi*_, *V*_*d*_ lysis rate	2.02·10^−9^	1.01·10^6^	1/*h*
*T*_0_	Initial target cell concentration	1.37·10^6^	−	*cells*/*mL*
*T*_*in*_	Cell concentration in feed	1.13·10^6^	−	*cells*/*mL*
*V*_*s*0_	Initial infectious STV concentration	3.16·10^5^	−	*virions*/*mL*
*V*_*ddi*0_	Initial DIP concentration	8.06·10^5^	−	*virions*/*mL*
*V*_*d*0_	Initial non-infectious STV concentration	2.03·10^7^	−	*virions*/*mL*

* Coefficient of variation (%) (Copasi)

** *Mean from experimental data of the virus bioreactors with μ = D, not fitted*.

The population of infected cells is subdivided into cells infected with infectious STVs (*I*_*s*_), DIPs (*I*_*d*_), and both (*I*_*c*_).

(5) $$\begin{array}{r} \frac{d\;I_{s}}{d\;t}=kvi\cdot T\cdot V_{s}-kvidi\cdot I_{s}\cdot V_{ddi}-\;kcdv\;\cdot I_{s}-D\cdot I_{s} \end{array}$$

(6) $$\begin{array}{r} \frac{d\;I_{d}}{d\;t}=kvidi\cdot T\cdot V_{ddi}-kvi\cdot I_{d}\cdot V_{s}-kcdv\cdot I_{d}-D\cdot I_{d} \end{array}$$

(7) $$\begin{array}{r} \frac{d\;I_{c}}{d\;t}=kvidi\cdot I_{s}\cdot V_{ddi}+kvi\cdot I_{d}\cdot V_{s}-\;kcdv\;\cdot I_{c}-D\cdot I_{c} \end{array}$$

The first term in Equations (5) and (6) accounts for the infection of target cells by infectious STVs or DIPs, respectively. Furthermore, the co-infection of *I*_*s*_ and *I*_*d*_ by DIPs and infectious STVs, respectively, yields co-infected cells *I*_*c*_ in Equation (7). Antiviral mechanisms, such as the interferon-mediated innate immune response, and virus-induced cell death can be triggered by the presence of intracellular viral RNAs. In our experiments, the uptake of a high number of DIPs (10^3^-10^4^ DIPs per cell) results in a high number of intracellular viral RNAs, comparable to levels reached during a conventional infection (e.g., Frensing et al., 2014). Therefore, and in contrast to Frensing et al. (2013) we assume that DIP-only infected cells (*I*_*d*_) do not continue to grow, but shut-off essential pathways for cell division, similar to the other infected cell populations *I*_*s*_ and *I*_*c*_ and, thus, die due to virus-induced apoptosis with the specific rate *kcdv*. Note, that Cane and colleagues have shown that DIP-only infected MDCK cells can continue to grow and give rise to DIP-infected daughter cells (Cane et al., 1987). However, the experimental conditions applied in their study, i.e., the use of UV-irradiated virus seed, passaging of cells in a virus-free culture and a culture time of 5-10 days between passages, cannot be compared to the cultivation conditions of our system.

The dynamics of infectious STVs (*V*_*s*_), DIPs (*V*_*ddi*_), and non-infectious virions containing a FL S1 (*V*_*d*_) are described as

(8) $$\begin{array}{rcl} \frac{d\;V_{s}}{d\;t} & = & \mu vi\cdot I_{s}-kvi\cdot T\cdot V_{s}-kvi\cdot I_{d}\cdot V_{s}-kdvit\cdot V_{s}\\ -D\cdot V_{s} \end{array}$$

(9) $$\begin{array}{rcl} \frac{dV_{ddi}}{d\;t} & = & \mu vddi_{C}\cdot I_{c}+\mu vddi_{S}\cdot I_{s}-kvidi\cdot T\cdot V_{ddi}\\ -kvidi\cdot I_{s}\cdot V_{ddi}-kvdt\cdot V_{ddi}\;-D\cdot V_{ddi} \end{array}$$

(10) $$\begin{array}{rcl} \frac{dV_{d}}{d\;t} & = & \mu vd_{S}\cdot I_{s}+\mu vd_{C}\cdot I_{c}+kdvit\cdot V_{s}-kvdt\cdot V_{d}\\ -D\cdot V_{d} \end{array}$$

Similar to Frensing et al. (2013), we assume that STV-infected cells (*I*_*s*_) produce infectious STVs (*V*_*s*_) with the specific rate μ*vi* and also have the potential to release DIPs (*V*_*ddi*_) with the specific rate μ*vddi*_*S*_. Although in our analysis μ*vddi*_*S*_ is close to zero (see Table 1), the latter should not be ignored to allow for *de novo* generation of DIPs in case a DIP-free virus seed would be used for the infection of bioreactors. Co-infected cells release mainly DIPs containing the deletion in S1 (*V*_*ddi*_) with the specific rate μ*vddi*_*C*_. In addition, both STV-infected and co-infected cells release non-infectious virus particles (V_*d*_) with rates μ*vd*_*S*_ and μ*vd*_*C*_, respectively. Furthermore, we assume that STVs are taken up by uninfected cells and DIP-only infected cells, while DIPs are either infecting uninfected cells or cells already infected by infectious STVs. Finally, we assume that STVs can lose their infectivity with the specific inactivation rate *kdvit* contributing to the population of non-infectious virions (*V*_*d*_), while both *V*_*ddi*_ and *V*_*d*_ deteriorate with the specific lysis rate *kdvt*. In contrast to the previous model (Frensing et al., 2013), we neglected superinfection of *I*_*s*_ and *I*_*c*_ by STVs as well as superinfection of *I*_*d*_ and *I*_*c*_ by DIPs.

To investigate the time course of DIP formation based on reverse transcription-qPCR measurements, the following DIP to STV ratio was determined from experimental data:

(11) $$\begin{array}{r} ratio=\;\frac{V_{ddi}}{V_{s}}, \end{array}$$

where *V*_*ddi*_ represent the reverse transcription-qPCR measurement of delS1(1) and *V*_*s*_ the infectious virions with FL S1, respectively. In addition, to describe overall cell growth dynamics, the concentration of all cells (*Cells*_*total*_) in the virus bioreactor was estimated.

(12) $$\begin{array}{r} Cells_{total}=\text{T}+I_{s}+I_{d}+I_{c} \end{array}$$

Taken together, the model comprises seven ordinary differential equations. The set of ten parameters (Table 1) was determined by minimizing the least-squares prediction error of the state variables *V*_*s*_, *V*_*ddi*_, *V*_*d*_ and *Cells*_*total*_, for which the error of each variable was weighted with its maximum measurement value.

Model equations were solved numerically using the CVODE routine from SUNDIALS (Cohen and Hindmarsh, 1996) on a Linux-based system. Model files and experimental data were handled within the Systems Biology Toolbox 2 (Schmidt and Jirstrand, 2006) for MATLAB (version 8.0.0.783 R2012b). Parameter values were estimated using the global stochastic optimization algorithm fSSm (Egea et al., 2007). Initial values were selected based on previous parameters determined for IAV replication in animal cells (e.g., Frensing et al., 2013). The coefficient of variation of parameters was determined using COPASI (Hoops et al., 2006).

## Results and Discussion

In order to reduce the risk of batch-to-batch variations for controlled experiments under different cultivation regimes, we established a parallel continuous production process for IAV propagated in MDCK suspension cells. In the set-up implemented, one cell bioreactor was used to feed two virus vessels (Figure 1). As a starting point, we investigated the impact of RT (22 and 36 h) on the accumulation of DIPs and their impact on viral titers over a period of 20 days. Based on quantitative information available from qPCR and the mathematical model established, it is possible to describe the dynamics of the various cell populations as well as virus populations, i.e., infectious STVs, non-infectious STVs and replication-incompetent DIPs, and the DIP to STV ratio for both cultivation conditions.

### Mathematical Model for Continuous Cultivation of Influenza A Virus

The mathematical model used in the present study is loosely based on a previously published model (Frensing et al., 2013) with modifications as explained in section Materials and Methods. We fitted the parameters of the model to the two sets of experimental data and determined their coefficient of variation (CoV) using Copasi (Table 1). For RT 22 h, low CoV (≤ 13%) were reached for parameter estimates of the specific growth rates for the different viral subpopulations *V*_*ddi*_ and *V*_*S*_, *V*_*d*_, which are released by either co-infected cells *I*_*c*_ (μ*vddi*_*C*_) or by STV-infected cells *I*_*S*_ (μ*vi*, μ*vd*_*S*_), respectively. Similarly, the CoV of estimates for μ*vddi*_*C*_, μ*vi* and μ*vd*_*S*_ for RT 36 h were ≤25%.

The specific STV infection rate *kvi* for RT 22 h as well as the specific DIP infection rate *kvidi*, and the specific apoptosis rate *kcdv* for both RT 22 and 36 h were estimated with higher CoV values (25% < CoV < 100%). Interestingly, the specific infection rate of STVs *kvi* for RT 36 h was estimated with a CoV of 15%.

The remaining parameters were estimated with CoV > 100%. In particular, the specific *de novo* generation rate of DIPs μ*vddi*_*S*_ had a very high CoV in both experimental scenarios. Since DIPs are present in the seed virus, *de novo* generation could also be excluded from modeling assumptions and viral dynamics would still show oscillations due to the amplification of DIPs introduced at infection. In contrast, if a DIP-free virus seed would be used to start infection, the oscillating viral dynamics could only be reproduced if *de novo* generation is accounted for. Thus, we decided to keep this parameter to propose a model that covers more general cases.

The specific production rate of non-infectious FL S1-containing virions μ*vd*_*C*_ could also be neglected, since these particles are most likely released only from STV-infected cells, which is supported by the reasonable CoV values for the specific *V*_*d*_ production rate (μ*vd*_*S*_) in both RT 22 and 36 h experiments. In addition, the high CoV values for estimates of the specific virus inactivation and lysis rates (*kdvit*, *kvdt*) indicated that these mechanisms can probably be neglected, which we also analyzed in more detail with respect to the different RTs (see section Model reduction).

Overall, key parameter values estimated in this study, such as specific STV infection rate *kvi*, virus release rates, apoptosis rate *kcdv* and virus degradation rates, were in the same order of magnitude as determined previously for IAV replication models proposed for adherent MDCK cells (Möhler et al., 2005; Schulze-Horsel et al., 2009; Heldt et al., 2013).

### Cellular Dynamics

**Figure 2:**
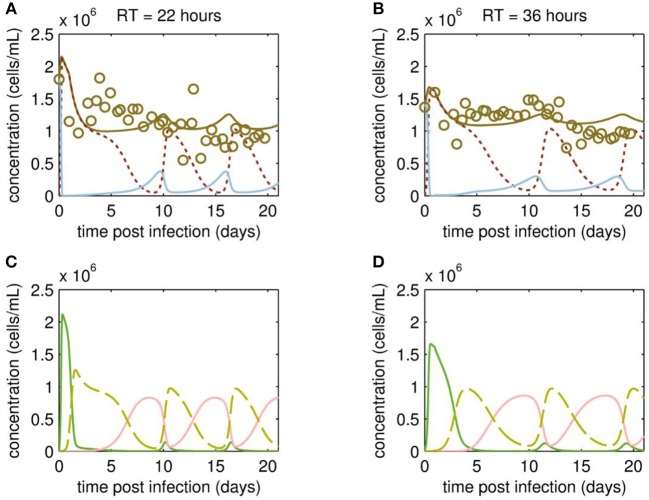
Cell population dynamics of MDCK.SUS2 cells infected by A/PR/8/34-delS1(1) in a parallel continuous bioreactor system at residence times (RTs) of 22 and 36 h. Measurements (open circles) and model fit (brown solid lines) of the total cell concentration, and the simulated number of all productively infected cells, i.e., the sum of STV-only infected and co-infected cells, as (dotted dark red line), and uninfected target cells (blue solid line) in the virus vessels for RTs of 22 h **(A)** and 36 h **(B)**, respectively. Model predictions for the different infected cell populations, cells infected by infectious STV only (green solid line), cells infected by DIP only (red solid line), co-infected cells (dashed yellow line) in the virus vessels are shown for RTs of 22 h **(C)** and 36 h **(D)**, respectively. For both, experiment and model simulations, the continuous culture was started 23.4 h p.i.

At first, MDCK suspension cells were seeded and grown in batch mode simultaneously in all three bioreactors CB, VB1 and VB2 (Figure 1). CB was set to a working volume of 1700 mL and cells were grown in batch mode until a cell concentration of 2.97·10^6^ cells/mL (viability 96.7 %) was reached. The infected vessels VB1 and VB2 had a working volume of 320 mL and 520 mL, respectively. VB1 and VB2 were seeded at a similar concentration, however, they reached 1.8·10^6^ cells/mL and 1.4·10^6^ cells/mL at time of infection, respectively. The infection of VB1 and VB2 took place at 50 h of culture at MOI of 0.1 and left in batch mode for another 23.4 h before initiating the continuous mode (to avoid virus washout). The VB1 and VB2 vessels were operated at an RT of 22 and 36 h, respectively. Those RTs were adjusted using a constant feed of 12 mL/h of cell broth from the CB and of 5.8 mL/h fresh medium into the virus bioreactors. Furthermore (to maintain wv), 17.8 mL/h were harvested from each VBs. The average concentration of target cells transferred from the CB to both VBs *T*_*in*_ was 1.13·10^6^ cells/mL. Upon parameter estimation, we simulated the cell population dynamics in the virus vessels, which showed oscillations for uninfected target cells and the various infected cell populations for both RT 22 and RT 36 h (Figure 2).

Although the total cell concentration in VBs remained stable with an average of 1.12·10^6^ cells/mL (standard deviation ± 0.30·10^6^ cells/mL) for RT 22 and 1.17·10^6^ cells/mL (standard deviation ± 0.22·10^6^ cells/mL) for RT 36 h, a tendency toward cyclic behavior was observed (Figures 2A,B). This is linked to the simulated dynamics of the various cell populations caused by virus propagation. As expected, addition of the virus seed causes a fast increase of STV-only infected cells, followed by a strong drop in their concentration and a simultaneous increase of the co-infected cell population through superinfection by DIPs (Figures 2C,D). The co-infected cell population reaches its maxima at ~2 days p.i. and 4 days p.i., for RT 22 and 36 h, respectively. Since co-infected cells release mainly DIPs, uninfected target cells fed into the VBs will be infected by DIP only. While the DIP-only infected cell population reaches a peak concentration at ~8 days p.i. (RT 22 h) and 10 days p.i. (RT 36 h), the population of co-infected cells undergoes virus-induced apoptosis and ceases to a minimum (Figures 2C,D). However, the number of co-infected cells rises again quickly, since previously DIP-only infected cells are now superinfected by STVs produced by the small sub-population of STV-only infected cells. This behavior is repeated for another 1 to 1.5 cycles within the cultivation time. Thereby, all subpopulations of infected cells reach their peak concentrations repeatedly, which are similar in both VBs and, thus, seem independent of the RT.

Overall, the variation in the measured cell concentrations in the VBs did not follow the oscillating trend described for similar experiments by Frensing et al. (2013) using the avian AGE1.CR cell line infected with IAV (A/Puerto Rico/8/34, MOI 0.025) for a RT of 25 h. Indeed, there are qualitative and quantitative discrepancies between model simulation and measurement of the total cell concentrations. On the one hand, counting of MDCK suspension cells required the addition of trypsin for dissociation of cell aggregates, which occurred occasionally and increased the risk of outliers in cell concentration measurements. On the other hand, our model is not as well-informed on the dynamics of the various infected cell subpopulations since measurements focused on viral dynamics (see following section Viral Dynamics). To better resolve this issue, follow-up studies are planned to investigate in detail the dynamics of the various cell populations using flow cytometry. Ideally, this would not only involve the conventional monitoring of infected cells using IAV-specific antibodies (Frensing et al., 2016), but the use of fluorescence markers for specific intracellular labeling of DI RNAs, which are currently not available, but under development and will allow to distinguish co-infected from STV-only infected cells.

### Viral Dynamics

**Figure 3:**
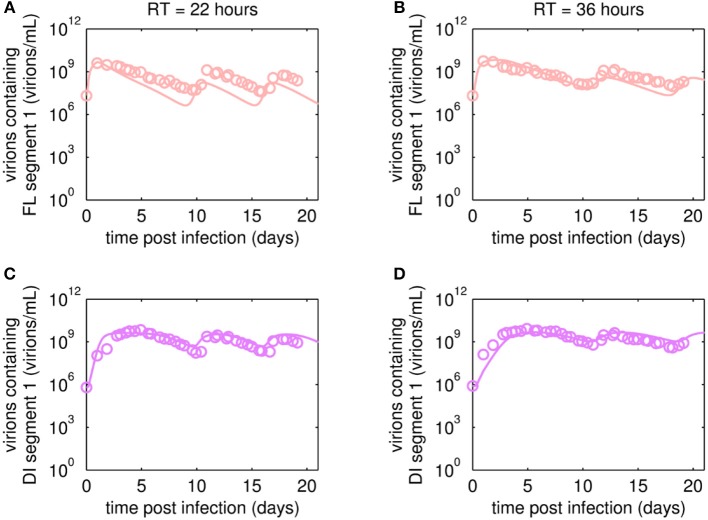
Dynamics of FL or DI segment 1 (S1)-containing virions of A/PR/8/34-delS1(1) produced in MDCK.SUS2 cells in a parallel continuous bioreactor system with residence times (RT) of 22 and 36 h, respectively. Experimental data based on reverse transcription-qPCR (open circles) and model fit (solid lines) are shown for **(A,B)** all virions containing FL S1 and **(C,D)** virions containing DI S1 (A/PR/8/34-delS1(1)). For both, experiment and model simulations, the continuous culture was started 23.4 h p.i.

**Figure 4:**
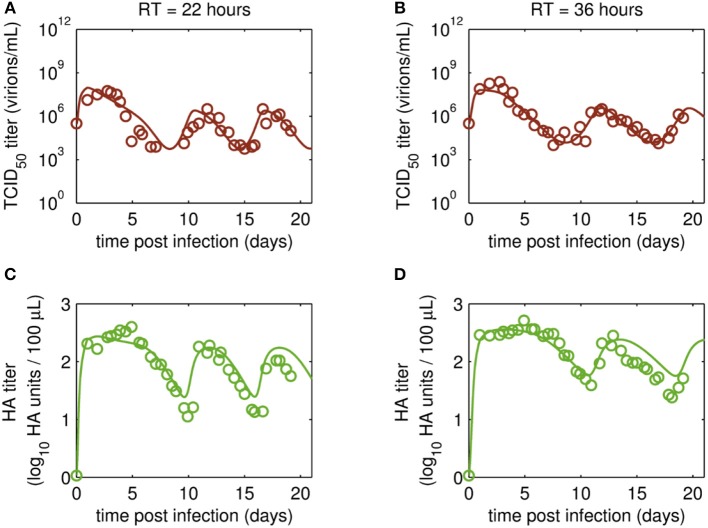
Dynamics of infectious virions and total number of virions produced in MDCK.SUS2 cells infected by A/PR/8/34-delS1(1) in a parallel continuous bioreactor system with residence times (RT) of 22 and 36 h, respectively. Experimental data (open circles) and model fit (solid lines) are shown for **(A,B)** TCID_50_ titer (infectious virion subpopulation of the FL S1-containing virions) and **(C,D)** HA titer which represents the sum of all viral subpopulations containing FL and DI S1. For both, experiment and model simulations, the continuous culture was started 23.4 h p.i.

The reverse transcription-qPCR measurements that enabled monitoring of both the FL S1 and DI S1 (A/PR/8/34-delS1(1)) copy numbers together with the concentrations of the total number of virus particles (HA titer) and TCID_50_ titer (Figures 3, 4), allowed discrimination of the various virus subpopulations. Using these values, we calculated the concentration of virions containing either FL or DI S1 based on the qPCR measurement given in “copies/mL” to “virions/mL” (see Equations 2 and 3).

Overall, viral titers of FL and DI S1-containing virions showed the same trend for the two RTs tested over the first 10 days of culture (Figure 3). Maximum concentrations of FL S1-containing virions with 4.0·10^9^ virions/mL and 5.6·10^9^ virions/mL were reached at ~1 day p.i. for RT of 22 and 36 h, respectively (Figures 3A,B). For RT 22 h, titers slowly decreased to about 10^7^ virions/mL at ~10 days p.i. For the higher RT, the decrease in titers of FL S1 virions was delayed compared to RT 22 and reached its minimum of about 10^8^ virions/mL at ~11 days p.i. At that time, the VB with RT 22 h already reached its second peak, followed by a decrease in titers leading to a second minimum at about 16 days p.i. The second minimum for RT 36 h was reached once more about 2 days later. The concentrations of DI S1-containing virions showed an initial delay in cycles of about 4 days compared to their corresponding FL counterparts and reached their first maxima with 7.0·10^9^ virions/mL (5 days p.i.) and 8.4·10^9^ virions/mL (5 days p.i.) for RT 22 and 36 h, respectively (Figures 3C,D). The cyclic trend observed in both data sets is described clearly by the model fits. Note, that some quantitative discrepancies between data and model simulation remain, such as underestimation of the FL S1-containing virus titers for various time points (Figures 3A,B). Although, measured and simulated virus titers show some discrepancies for these individual runs, simulations are mainly within the error range of the assays for HA (about ± 0.15 log) and TCID_50_ (about ± 0.3 log). Regarding the error for the number of virions containing either FL or DI S1, it has to be considered that those are derived from both qPCR measurements (relative standard deviation of about 25%) and from HA titers (Equations 2 and 3). Nevertheless, further investigations (e.g., next-generation deep sequencing) should be performed to elucidate those discrepancies in more detail.

Time courses for infectious virions *V*_*s*_ measured by the TCID_50_ assay and for the sum of all viral subpopulations determined by the HA assay (*V*_*s*_+*V*_*d*_+*V*_*ddi*_) also showed pronounced oscillations (Figure 4). Furthermore, the HA data indicate a trend for an overall decrease in the amplitude of titers toward later cultivation time points for both RTs (Figures 4C,D). The TCID_50_ titers reached their maxima with the initial peaks, while the following peak titers were lower. However, no overall decrease in peak infectious titers was observed (Figures 4A,B).

Maximum TCID_50_ titers were achieved around 3 days p.i. with 5.6·10^7^ virions/mL and 2.4·10^8^ virions/mL for RT 22 and 36 h, respectively. Most likely, the higher infectious titer for the longer RT of 36 h was related to the reduced wash-out (lower dilution rate) compared to RT 22 h. In total, three cycles were observed for the short RT with a second and a third peak of ~3·10^6^ virions/mL at 12 and 18 days p.i., respectively (Figure 4A). A similar behavior was observed for RT 36 h, however, only about 2.5 cycles were achieved during the cultivation time (Figure 4B). While the TCID_50_ dynamics are captured well both quantitatively and qualitatively, simulated and measured HA values deviate to a certain extent (but within the assay error range) for data points after about 10 days p.i. (Figures 4C,D). In particular, the decreasing trend of peak HA titers is not reproduced by the model (also in case the system is simulated for 90 days p.i., Data Sheet 1; Figure S1). Whether the decreasing trend of peak HA titers is an individual characteristic of these particular runs or if the model simulations still lie within the biological variation of these experiments needs to be addressed when more experiments become available. Since the overall goal of this study was to establish and fit a basic model with a minimum number of parameters and simple kinetics, we decided to keep the model structure for now. Likely, also more detailed studies regarding the dynamics of the individual cell populations will allow further improvement of the model (see discussion on *Cellular dynamics*).

### Simulation of DIP to STV Ratios

**Figure 5:**
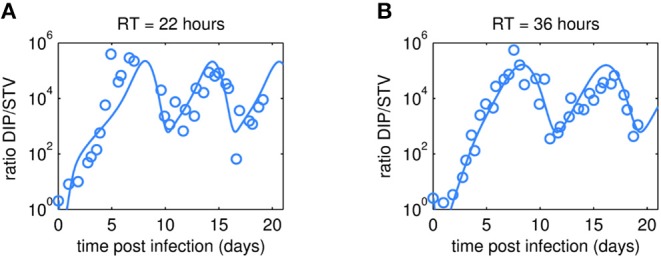
Dynamics of DIP to STV ratios observed in MDCK.SUS2 cells infected by A/PR/8/34-delS1(1) in a parallel continuous bioreactor system at residence times (RT) of 22 h **(A)** and 36 h **(B)**. The experimental DIP to STV ratio determined by dividing the DI S1 containing number of virions by the corresponding TCID_50_ titer (circles) is shown together with the simulated ratio according to Equation (11).

As expected, oscillations were also observed in the dynamics of the DIP to STV ratios (Figure 5). In both cultivations, the ratio reached a maximum of about 10^5^ at ~7 days p.i. These maxima are similar for both RTs and correlate with the lowest TCID_50_ titer. We may hypothesize that one infectious STV per 10^5^ DIPs is a critical ratio at which DIPs cause self-interference and start to hamper overall virus replication significantly. The impact of too high DIP to STV ratios and related self-interference is supported by a modeling study of our group (Laske et al., 2016). As a consequence of self-interference, DIP concentrations decrease, reaching a minimum around 11 days p.i., and numbers of infectious STVs are rising again, initiating a new cycle (see also Figures 3, 4). It would be interesting to test whether this critical ratio can be reproduced in other experiments of continuous two-stage cultivations.

### Model (In)Validation

**Figure 6:**
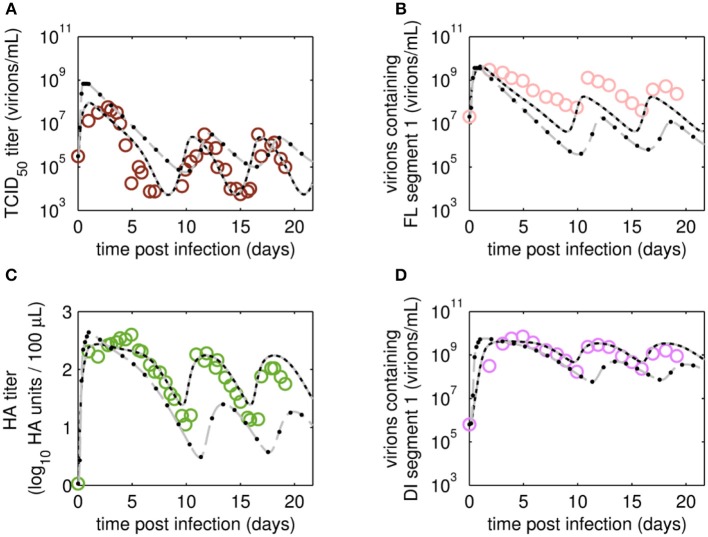
Dynamics of viral subpopulations, produced by MDCK.SUS2 cells infected by A/PR/8/34-delS1(1) in a parallel continuous bioreactor system at RT 22 h, were fitted using different models. Experimental data (open circles) and four model fits (various lines) are shown for **(A)** TCID_50_ titer representing infectious virions of **(B)** the FL S1-containing virions as well as the sum of all viral subpopulations as log HA units **(C)** and DI S1-containing virions **(D)**. Every subfigure shows the four model fits, using Model 1 containing all parameters (gray solid line), Model 2 with *kdvit* = *kvdt* = 0 (black dotted line), Model 3 with *kvi* = *kvidi* (gray dashed line) or Model 4 with *kvi* = *kvidi* and *kdvit* = *kvdt* = 0 (line of black circles). For the sake of simplicity only every 2^nd^ data point is shown for the qPCR-based data **(B,D)**. For both experiment and model simulations, the continuous culture was started 23.4 h p.i.

**Figure 7:**
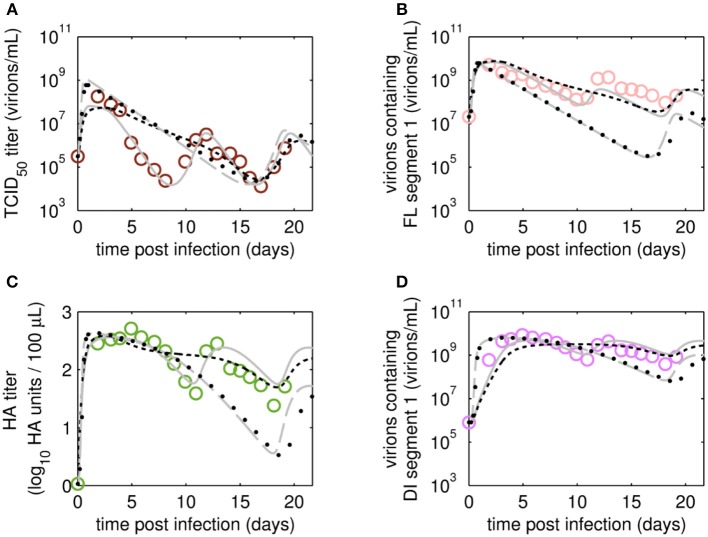
Dynamics of viral subpopulation, produced by MDCK.SUS2 cells infected by A/PR/8/34-delS1(1) in a parallel continuous bioreactor system at RT 36 h, were fitted using different models. Experimental data (open circles) and four model fits (various lines) are shown for **(A)** TCID_50_ titer representing the infectious virions of **(B)** the FL S1-containing virions as well as the sum of all viral subpopulations as log HA units **(C)** and DI S1-containing virions **(D)**. Every subfigure shows the four model fits, using Model 1 containing all parameters (gray solid line), Model 2 with *kdvit* = *kvdt* = 0 (black dotted line), Model 3 with *kvi* = *kvidi* (gray dashed line) or Model 4 with *kvi* = *kvidi* and *kdvit* = *kvdt* = 0 (line of black circles). For the sake of simplicity, only every 2^nd^ data point is shown **(A–D)**. For both, experiment and model simulations, the continuous culture was started 23.4 h p.i.

After fitting the present model (“Model 1”) to both data sets, we tested if it could still describe the data if certain parameters were removed. First, we set the parameter for inactivation of *V*_*s*_ (*kdvit*) and the lysis rate of virions (*kvdt*) to zero and re-fitted the model (“Model 2”). For the data set of RT 22 h, the goodness of fit was similar to the full model (Model 1), both visually (Figure 6) and quantitatively as based on the objective function values (Table 2). This was expected, since both parameters, *kdvit* and *kvdt*, were already close to zero in Model 1. For the second data set with RT 36 h, the re-fitting with Model 2 resulted in a noticeable increase of the objective function value (Table 3). In addition, Model 2 was unable to describe the data qualitatively. In particular, the number of cycles in the viral dynamics could not be reproduced any more (Figure 7). Most likely this points to an important feature of cultivations with long RTs, for which degradation and inactivation processes have to be taken explicitly into account to adequately describe the data. In contrast, in cultivations with short RTs (higher dilution rates), the overall impact of the wash-out of virions on viral dynamics was probably higher than that of inactivation and degradation processes.

**Table 2:** Parameterization and objective function value obtained by fitting different models for the production of A/PR/8/34-delS1(1) by MDCK.SUS2 cells in a parallel continuous bioreactor system at residence time 22 h.

**Parameters**	**Model 1, all parameters**	***Model 2, kdvit* = *kvdt* = 0**	***Model 3, kvi* = *kvidi***	***Model 4, kdvit* = *kvdt* = 0 and *kvi* = *kvidi***
				
*kvi*	1.59·10^−7^	1.59·10^−7^	4.58·10^−9^	4.58·10^−9^
*kvidi*	2.32·10^−10^	2.33·10^−10^	*kvi*	*kvi*
*kcdv*	0.008	0.008	0.009	0.009
μ*vi*	2.51	2.50	105	104
μ*vddi*_*C*_	203	203	168	168
μ*vddi*_*S*_	1.00·10^−5^	1.15·10^−5^	0.01	0.01
μ*vd*_*C*_	4.91·10^−16^	3.13·10^−16^	2.11·10^−13^	2.82·10^−13^
μ*vd*_*S*_	120	120	439	441
*kdvit*	1.58·10^−7^	0	1.58·10^−4^	0
*kvdt*	3.82·10^−27^	0	6.30·10^−26^	0
Objective function value^#^	8.50	8.49	7.99	8.00

# *Objective function values are the normalized least squared prediction errors of the state variables of all cells Cells_total_, fully infectious STVs V_S_, replication-incompetent DIPs V_ddi_, and non-infectious FL S1-containing virions V_d_*.

**Table 3:** Parameterization and objective function value obtained by fitting different models for the production of A/PR/8/34-delS1(1) by MDCK.SUS2 cells in a parallel continuous bioreactor system at residence time 36 h.

**Parameters**	**Model 1, all parameters**	***Model 2, kdvit* = *kvdt* = 0**	***Model 3, kvi* = *kvidi***	***Model 4, kdvit* = *kvdt* = 0 and *kvi* = *kvidi***
				
*kvi*	5.38·10^−8^	1.38·10^−7^	2.89·10^−9^	2.60·10^−9^
*kvidi*	7.96·10^−11^	1.16·10^−10^	*kvi*	*kvi*
*kcdv*	0.003	0.002	0.001	0.001
μ*vi*	4.12	1.42	142	80
μ*vddi*_*C*_	177	93	152	153
μ*vddi*_*S*_	1.13·10^−9^	2.57·10^−7^	1.04·10^−10^	1.62·10^−7^
μ*vd*_*C*_	1.41·10^−8^	1.42·10^−9^	1.01·10^−5^	2.01·10^−6^
μ*vd*_*S*_	173	193	613	725
*kdvit*	0.07	0	4.35·10^−3^	0
*kvdt*	2.02·10^−9^	0	2.15·10^−8^	0
Objective funciton value^#^	6.19	13.36	4.99	5.06

# *Objective function values are the normalized least squared prediction errors of the state variables of all cells Cells_total_, fully infectious STVs V_S_, replication-incompetent DIPs V_ddi_, and non-infectious FL S1-containing virions V_d_*.

Next, we tested whether it is essential to account for separate specific rates of infection, for either STV (*kvi*) or DIP infection (*kvidi*). For this, we used Model 1 and defined one joint infection rate (*kvi* in “Model 3”). Upon fitting Model 3 to the data of RT 22 h, the overall dynamics were still described well. However, the model overestimated the TCID_50_ titer while underestimating the number of FL and DI S1-containing virions as well as the overall number of virions produced (Figure 6). We observed a similar effect for the RT 36 h data set, combined with a lower number of cycles compared to the fit with Model 1 (Figure 7). This suggests that two separate infection rates are needed to achieve good agreement with experimental data. Although, visually, goodness of fit with Model 1 in both data sets seems better than that of Model 3, the objective function value has decreased only by 6% or by 19% compared to that of Model 1, for the RT 22 h (Table 2) and RT 36 h (Table 3), respectively. At a first glance, this decrease in the objective function value seems counter-intuitive, however, since Model 3 still has a very good agreement with a majority of measurements for some of the state variables, e.g., DIP concentration (Figure 7D), the objective function value might be comparable to that of Model 1. While fitting oscillating data sets, we experienced that the objective function value can be misleading for some parameter regimes and that, for instance, optimizers may also yield “a good quantitative fit” by a straight line through the mean of the experimental data.

Finally, we also tested a fourth version of the model, which consisted of a combination of Model 2 and Model 3, i.e., neglecting virus degradation processes and using a joint infection rate, simultaneously. In case of RT 22 h, the Model 4 followed the same dynamics as Model 3 (Figure 6). This was expected, since the analysis of Model 2 already showed that degradation processes are not highly relevant for RT 22 h. Thus, Model 3 and Model 4 are mechanistically identical, which also leads to similar parametrizations of these two models (Table 2). For RT 36 h, the dynamics of Model 4 were similar to Model 2, showing fewer cycles due to exclusion of virus inactivation and degradation processes (Figure 7). In addition, Model 4 showed quantitative deviations similar to those of Model 3. Together, this underlines the importance of virus inactivation and degradation processes as well as the separation of DIP and STV infection rates for RT 36 h, which have to be taken into account to capture the experimental data. Most likely the difference in *kvi* and *kvidi* is related to the underlying mass action kinetics of the model. Since the DIP concentration is, on average, about two orders of magnitude higher than the STV concentration, the model estimates a lower specific DIP infection rate to yield an adequate amount of DIP-only and co-infected cells, and a high DIP titer in the supernatant. Whether this has any biological background still needs to be addressed experimentally.

Ideally, models including parametrization should allow the prediction of viral dynamics for different RT, e.g., to perform model-based process optimization. However, due to some mechanisms that seem dependent on the RT (explained above) none of the current model-parameter-combinations was able to predict viral dynamics for the other RT and *vice versa* (see also Data Sheet 1; Figures S2, S3). Accordingly, we think that the model might still lack certain aspects or kinetics and therefore has room for further model extensions. For instance, including the eclipse phase, i.e., the time delay between virus infection and release of viral progeny, or taking into account the accumulation of other DI RNAs, except for A/PR/8/34-delS1(1), might help to further improve model fits and enable predictions (see Data Sheet 1; Figure S4).

## Summary

In the present study, we introduce a two-stage continuous bioreactor setup for head-to-head comparison of IAV DIP production under different culture conditions. For infection, we used a virus seed generated by reverse genetics, which contained a known DIP, A/PR/8/34-delS1(1), which enabled the monitoring of DI and FL virus replication based on qPCR measurements. We observed oscillations in viral titers, where the frequency was depending on the RT. I.e., an increase in the RT from 22 to 36 h caused a shift in cycles of about 2 days. PCR analysis of IAV segments 1-3 revealed that changes in the RT might also result in the accumulation of different DIP subpopulations in long-term cultures. The mathematical model established allowed to describe the time courses of the various viral and cellular subpopulations. Comparison of model fits to the two data sets obtained suggests that for the longer RT of 36 h, inactivation of infectious STVs and degradation of virions has to be taken into account. Interestingly, the goodness of fit was also affected by the additional assumption that DIPs and STVs infect cells at different rates. Still, we observed some discrepancies between model and experimental data and found that the predictive power of the model needs improvement. This may be related to the fact that the model is still not fully informed, e.g., regarding the dynamics on the different infected cell subpopulations and the *de novo* synthesis and impact of other DIPs except for A/PR/8/34-delS1(1). In addition, the model might be extended further by accounting for the eclipse phase of virus release. The use of reporter viruses and specific probes for DI RNA staining may help to elucidate those mechanisms in more detail. For parameter estimation, we have used the data set of only one parallel run and are, therefore, unable to evaluate goodness of fit with respect to the biological variation of these cultivations. This will be addressed in the future, when more cultivations performed at similar experimental conditions become available.

In summary, the continuous cultivation system established is an excellent tool for detailed studies regarding DIP replication in animal cells. Based on the high DIP concentrations obtained it also qualifies as a system for production of DIPs for animal trials and influenza antiviral therapy.

## Data Availability Statement

All datasets generated for this study are included in the manuscript/Supplementary Files.

## Author Contributions

FT, TL, MW, MR, YG, and UR contributed conception and design of the study. FT performed the experiments. TL, MR, and UR performed the mathematical analysis. UR wrote the first drafts of the manuscript. FT, TL, MW, YG, and UR wrote sections of the manuscript. All authors contributed to manuscript revision, read, and approved the submitted version.

### Conflict of Interest

The authors declare that the research was conducted in the absence of any commercial or financial relationships that could be construed as a potential conflict of interest.
