# Supplementary material for: Production of Defective Interfering Particles of Influenza A Virus in Parallel Continuous Cultures at Two Residence Times—Insights From qPCR Measurements and Viral Dynamics Modeling
Source: Front Bioeng Biotechnol. 2019 Oct 18;7:275. doi: 10.3389/fbioe.2019.00275 (PMC6813217; doi:10.3389/fbioe.2019.00275)
Supplement: Supplementary file 1 [file Data_Sheet_1.docx]

Supporting information


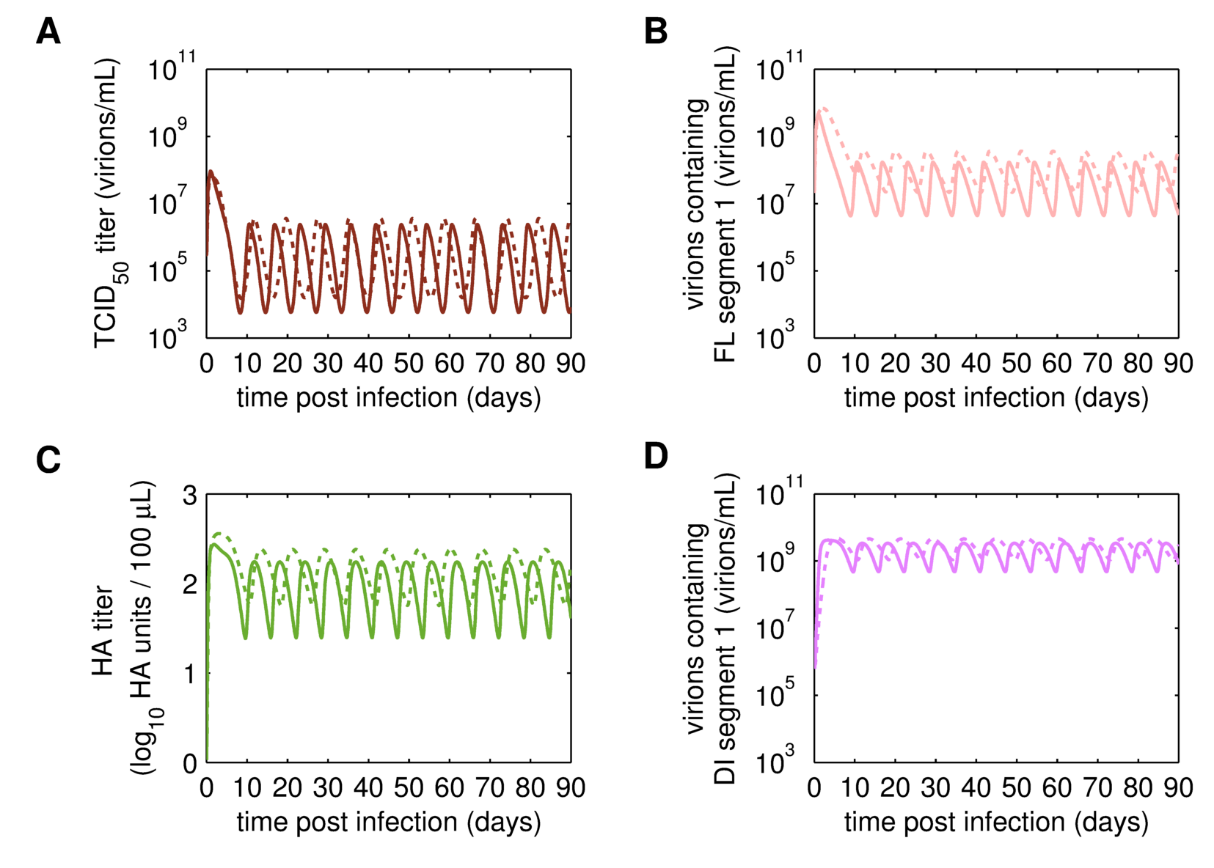


**S1 Figure**: **Dynamics of viral subpopulations of A/PR/8/34-delS1(1), produced by MDCK.SUS2 cells in a parallel continuous bioreactor system at residence times of 22 h and 36 h over 90 days.** Time courses of infectious STVs (A), FL S1-containing virions (B), sum of all viral subpopulations in log HA units (C), and DI S1-containing virions (D) are shown for residence times of 22 h (solid lines) and 36 h (dashed lines).

Supporting discussion: Model predictions

So far, we performed parameter fits to RT 22 h and RT 36 h, resulting in two parameter sets for the same experimental setup. It would be reasonable to expect that the model established can reproduce viral dynamics for multiple RTs. Therefore, we tested whether the full model (Model 1) for one RT could be used to predict the viral dynamics for the other RT by adjusting only the dilution rate. First, we used Model 1 calibrated to the data set of RT 22 h to predict the viral dynamics of the continuous cultivation running at RT 36 h. For this, we applied the corresponding modified RT by setting the parameter $\mu$ = D = 0.027 1/h, simulated the model and compared it to the data (S2 Figure). The model simulation still showed oscillations in the viral dynamics. The maxima and minima were predicted in reasonable agreement to the measurements for the TCID_50_ titer (S2 Figure A), total number of virions (S2 Figure C) and number of DI S1 containing particles (S2 Figure D). However, the number of cycles was not in agreement to the experimental data. Second, we also predicted viral dynamics for the cultivation running at RT 22 h using Model 1 calibrated against the data set obtained for RT 36 h. Upon setting $\mu$ = D = 0.045 1/h, the model simulation did not show any oscillations (S3 Figure). Taken together, Model 1 neither trained with the data set of RT 22 h nor with that of RT 36 h can make reasonable predictions for other residence times. We hypothesize that this is related to some underlying mechanisms that are characteristic to either short or long residence times, for instance, differences in the impact of virion inactivation and degradation on viral dynamics (see Section on Model reduction). Attempts to fit the model to both data sets simultaneously also failed. Based on a more comprehensive set of experiments and an extension of the model, it might be possible to train a “master model” with various data sets simultaneously and find a joint set of parameters that allows predictions for a wider range of residence times.

In addition, our model might not be detailed enough to capture all aspects of virus growth relevant for different residence times. In particular, we imagine that the eclipse phase, i.e., the time delay between virus infection and onset of viral progeny release, needs to be taken into account. In a scenario where low concentrations of infecting viruses are reached, i.e., minima of the cycles, and target cells are infected at a low MOI, the eclipse phase may have a strong impact on the onset of the following cycle. In addition, we made the simplifying assumption that DI S1 particles are the only DIPs interfering in the cultivations. However, it may well be that other DI RNAs, which arise *de novo* influence viral dynamics through additional interference (see Supporting discussion: Segment-specific reverse transcription-PCR, S4 Figure). Since we cannot yet identify and quantify such DIPs, we excluded corresponding kinetics from the model as we expected difficulties in identification of kinetic parameters related to their replication.


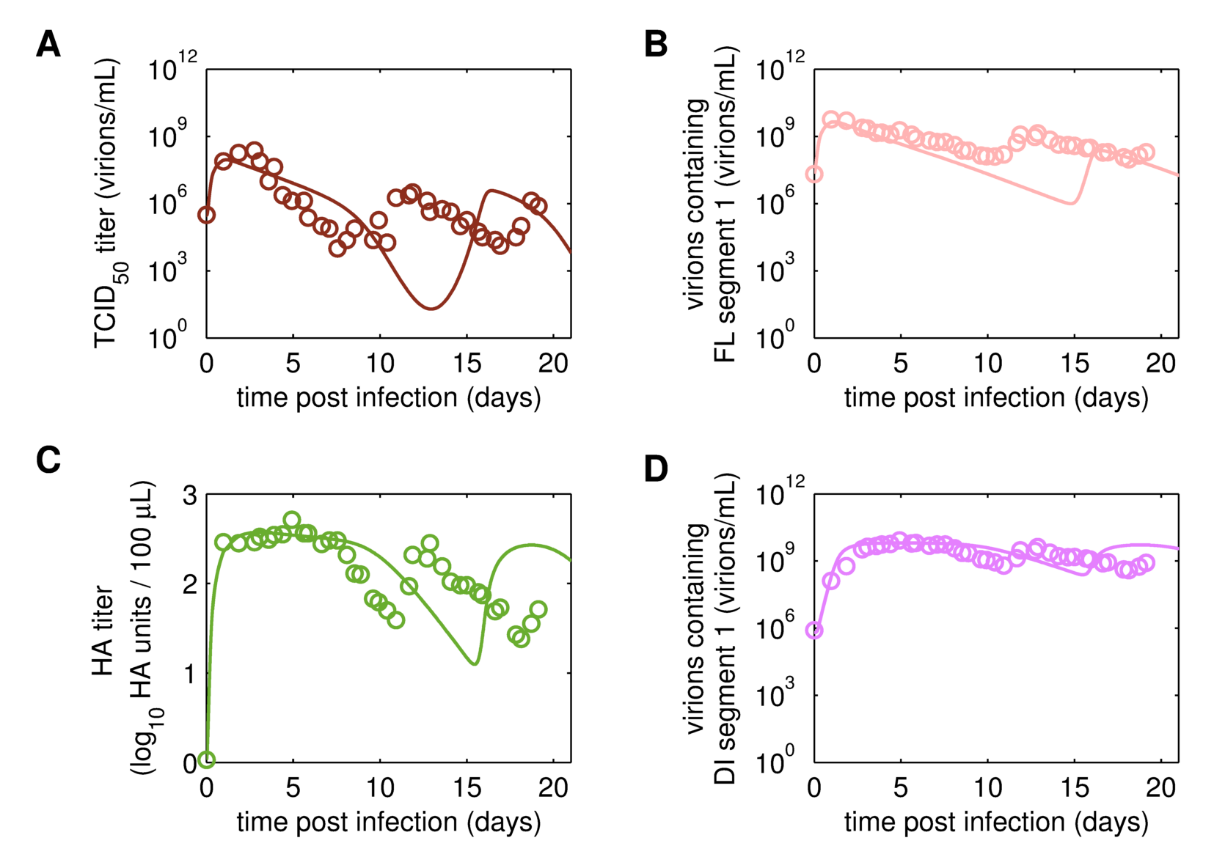


**S2 Figure:** **Prediction for the dynamics of viral subpopulations of A/PR/8/34-delS1(1), produced by MDCK.SUS2 cells in a parallel continuous bioreactor system at a residence time (RT) of 36 h**. Experimental data (open circles) of RT 36 h and predictions (lines) using the model previously fitted to data of RT 22 h with a change to $\mu$ = D = 0.027 1/h. Shown are (A) TCID50 titer representing the infectious virions of (B) the FL S1-containing virions as well as the sum of all viral subpopulations as log HA units (C) and DI S1-containing virions (D). For both, experiment and model simulations, the continuous culture was started 23.4 h p.i.


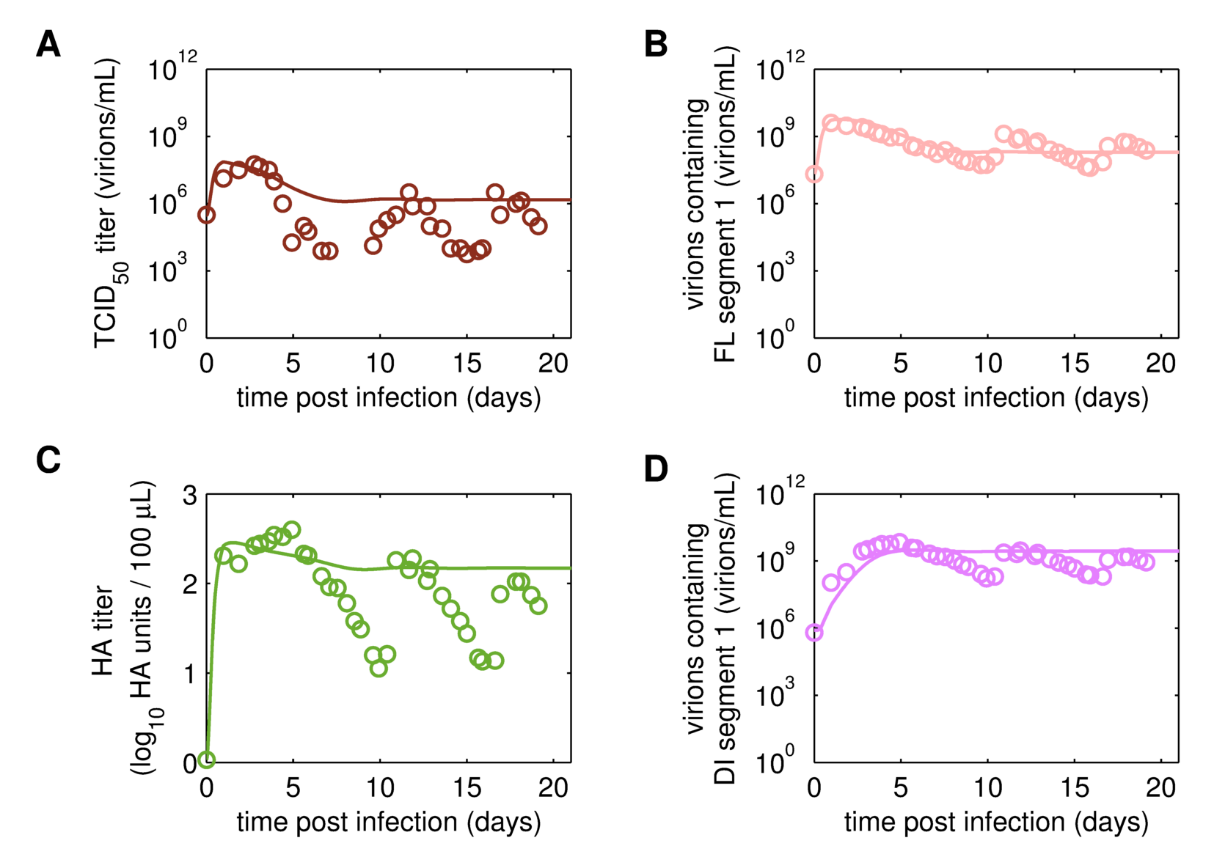


**S3 Figure**: **Prediction for the dynamics of viral subpopulations of A/PR/8/34-delS1(1), produced by MDCK.SUS2 cells in a parallel continuous bioreactor system at a residence time (RT) of 22 h**. Experimental data (open circles) for a RT of 22 h and predictions (lines) using the model fitted to data of RT 36 h with a change to $\mu$ = D = 0.045 1/h. Shown are (A) TCID_50_ titer representing the infectious virions of (B) the FL S1-containing virions as well as the sum of all viral subpopulations in log HA units (C) and DI S1-containing virions (D).

Supporting discussion: Segment-specific reverse transcription-PCR

Accumulation of defective segments (DS), including DI segments and non-interfering deleted segments, in IAV was evaluated from bioreactor samples using a segment-specific PCR. Influenza virus segments 1-3, encoding PB2, PB1 and PA, are known to accumulate more DS than other segments in continuous passaging (Frensing et al., 2013). Hence, differences in DS accumulation in segments 1-3 at two RTs were evaluated. In both cultivations, the presence of A/PR/8/34-delS1(1) virus with about 500 base pairs (bp) was detected as expected (left and right, S4 Figure). In general, DS in the range of 500-1000 bp were detected in all three segments and for both RTs. Oscillations of DS were observed in both RT experiments as noticeable by changes in the brightness of the bands over cultivation time. For example, in the 22 h RT experiment, a group of sharp and bright bands was followed by faint bands at about 8 and 16 days p.i., which coincide with the lowest TCID_50_ titers in both vessels. This periodicity can be observed more easily in the 22 h RT culture due to the low HA and TCID_50_ titers obtained at the end of each oscillation as a consequence of a higher dilution rate. Interestingly, in all three segments, a tendency towards generation of more DS in the range 500-1000 bp was observed in the 22 h RT culture compared to the 36 h RT. In particular, segment 3 showed a different pattern among RTs in the range of 500-1000 bp between 12 and 18 days p.i. Here, a double band of DS was observed in the 22 h RT, but not in the 36 h RT experiment. Finally, this result suggests that different RTs in long-term continuous cultures may lead to the generation of different DIP subpopulations and that continuous cultivations performed over a wider range of RT could be useful for virus evolution studies (Domingo and Perales, 2018; Gregori et al., 2016).


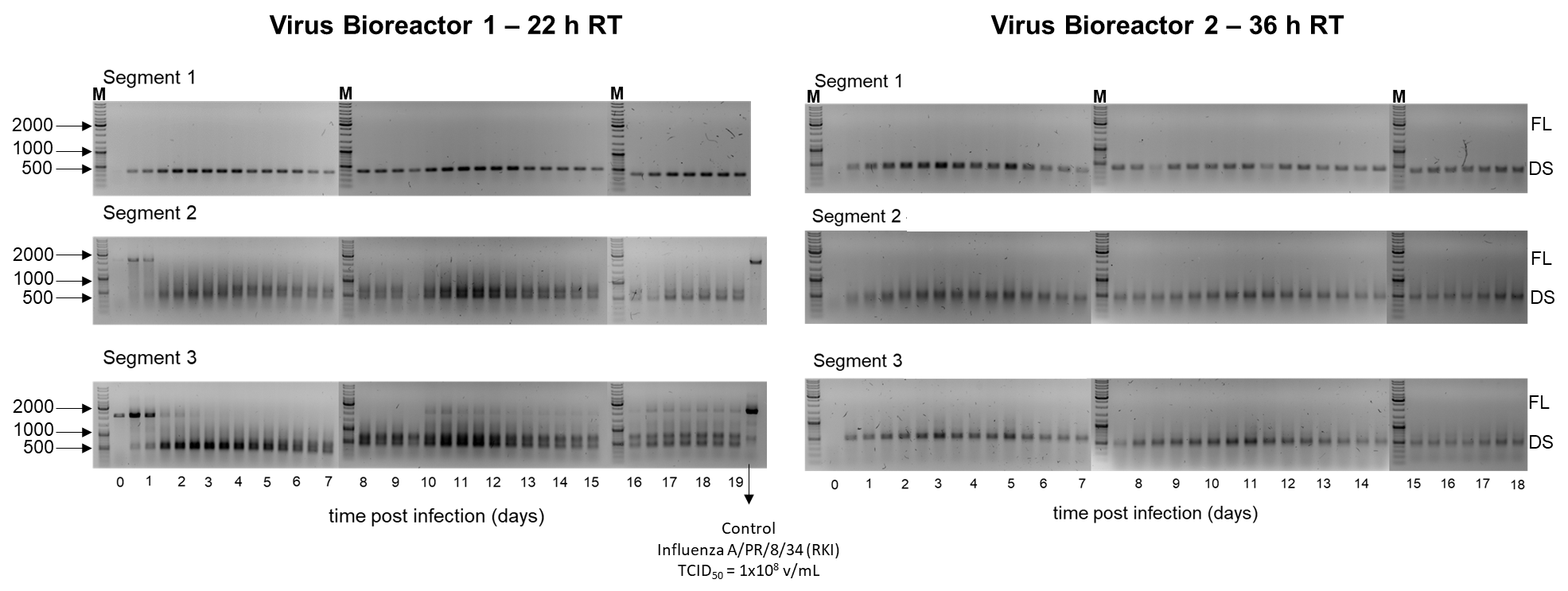


**S4 Figure. Segment-specific reverse transcription-PCR for detection of influenza A defective genomes propagated in a parallel continuous bioreactor system at two residence times (RT).** Full-length (FL) of about 2000 base pairs (bp) and defective segments (DS) of 500-700 bp were amplified (Frensing et al., 2014) and identified by marker bands (M) at 2000, 1000 and 500 bp. Left: reverse transcription-PCR of segments 1, 2 and 3 of influenza A virus in virus bioreactor 1 with 22 h RT. Right: reverse transcription-PCR of segments 1, 2 and 3 of influenza A virus in virus bioreactor 2 with 36 h RT. The control sample using influenza A/PR/8/34 is included in the virus bioreactor 1 gel
